# Modulation of individual and synchronized activities of ankle plantarflexors during quiet standing in aroused emotions

**DOI:** 10.1007/s00221-025-07046-3

**Published:** 2025-04-22

**Authors:** Ryogo Takahashi, Naotsugu Kaneko, Atsushi Oshima, Naoki Tsukamoto, Bowen Liu, Inhyeok Jeong, Mayu Dohata, Kimitaka Nakazawa

**Affiliations:** 1 Department of Life Sciences, Graduate School of Arts and Sciences, The University of Tokyo, Tokyo, Japan; 2 Japan Society for the Promotion of Science (JSPS), Tokyo, Japan

**Keywords:** Postural control, Standing, Electromyogram, Intermuscular coherence, Emotion, Autonomic nervous system

## Abstract

Emotions influence postural control, but previous studies have mainly examined postural dynamics during quiet standing using the center of pressure as a measure. During quiet standing, neuromuscular activities of ankle plantarflexors are crucial for maintaining postural balance, both in terms of individual and synchronized activities. This study aimed to clarify the emotional effects on the individual and synchronized neuromuscular activities of ankle plantarflexors during quiet standing. Twenty-four healthy male participants were instructed to stand and view emotional pictures for 72 s following a fixation cross. The task was repeated four times with four picture conditions, which was composed of two arousals (High and Low) and two valences (Pleasant and Unpleasant). During the task, electromyograms (EMG) of the tibialis anterior (TA), soleus (SOL), and medial (MG) and lateral gastrocnemius muscles (LG) were recorded. The EMG signals were rectified, and mean amplitude was calculated to assess individual neuromuscular activity. Inter-muscular coherence (IMC) between ankle plantarflexors was calculated to assess synchronized neuromuscular activity, with mean IMC calculated at 0–4 Hz, 8–12 Hz, 15–30 Hz, and 30–40 Hz. Results showed that aroused emotions induced a reduction in SOL activity. Aroused emotions also increased IMC at 8–12 Hz, indicating enhanced physiological tremor and reduced capacity for postural adjustment. In addition, IMC at 15–30 Hz was increased while 30–40 Hz was decreased in aroused emotions, implying modulation of cortical common drive to ankle plantarflexors.

These findings deepen our understanding of the emotional impacts on standing postural control.

**Supplementary Information:**

The online version contains supplementary material available at 10.1007/s00221-025-07046-3.

## Introduction

Upright standing is a fundamental posture essential for daily life. A decline in standing balance increases the risk of falling and restricts many physical and social activities (Hartholt et al. 2011; Ambrose et al. 2013; Dijkstra et al. 2020). Therefore, exploring factors that affect standing balance is crucial for improving the quality of life. Recent studies have provided evidence that standing balance is influenced by emotional factors, as measured by the center of pressure (COP) while participants viewed emotion-eliciting pictures (Azevedo et al. 2005; Facchinetti et al. 2006; Stins and Beek 2007; Roelofs et al. 2010; Horslen and Carpenter 2011; Mouras et al. 2015; Ciria et al. 2017; Kordts-Freudinger et al. 2017; Takahashi et al. 2024). Emotional states are generally expressed using a two-dimensional model consisting of arousal and valence. Arousal indicates the intensity of the emotion (high/low), while valence represents the level of pleasantness (pleasant/unpleasant). In previous studies examining emotional effects on postural control, this arousal-valence model has been used to manipulate participants’ emotional states (Azevedo et al. 2005; Facchinetti et al. 2006; Stins and Beek 2007; Roelofs et al. 2010; Horslen and Carpenter 2011; Mouras et al. 2015; Ciria et al. 2017; Kordts-Freudinger et al. 2017; Takahashi et al. 2024). For example, Horslen and Carpenter found that high-arousal emotions led to more frequent COP sway (i.e., mean power frequency: MPF) compared to low-arousal emotions, regardless of valence level (Horslen and Carpenter 2011). In contrast, our recent study showed that unpleasant-valence emotions induced more frequent (i.e., MPF) and smaller amplitude (i.e., standard deviation) of the COP than pleasant-valence emotions, independent of arousal level (Takahashi et al. 2024). Considering that the COP reflects ankle torque and is driven by ankle dorsiflexor or plantarflexor activities (Masani et al. 2003), it is crucial to examine how arousal- and valence-dependent changes in the COP are linked to the neuromuscular modulation of these muscles.

Recently, we investigated the effects of arousal and valence on the neuromuscular activities of ankle dorsiflexor (i.e., tibialis anterior muscle, TA) and plantarflexor (i.e., soleus muscle, SOL) in young healthy males during quiet standing using electromyogram (EMG) (Takahashi et al. 2024). Our results showed no significant arousal or valence effect on the neuromuscular activities of the TA and SOL, despite the changes in COP associated with valence level. However, this study lacked physiological validation of arousal manipulation, such as electrodermal activity, although heart rate successfully indicated valence manipulation. This raises the possibility that the significant valence effects on postural control were contaminated by potential arousal effect. In fact, exposure to a height environment (i.e., postural threat) modulates TA, SOL, and medial gastrocnemius muscle (MG) activities in parallel with arousal levels (Carpenter et al. 2001a) with recent evidence highlighting its impact on neural circuits driving SOL activity (Zaback et al. 2022). Therefore, the possibility cannot be eliminated that the activities of ankle dorsiflexor and plantarflexors, including TA, SOL, and MG, are modulated by arousal during quiet standing.

In addition to individual neuromuscular activities, the present study also focused on synchronized neuromuscular activity between ankle plantarflexors, which represents a fundamental neural mechanism underlying postural control in quiet standing (Obata et al. 2014; Nandi et al. 2019; Nojima et al. 2020). The synchronized activity between multiple muscles originates from common neural inputs to different spinal motoneuron pools (Boonstra et al. 2008a). Synchronized activity between the ankle plantarflexors during quiet standing has been reported to contain two main distinct frequency components: 0–4 Hz and 8–12 Hz (Obata et al. 2014). The former frequency band (0–4 Hz) reflects postural sway-related synchronized activity, which may explain changes in COP due to emotional modulation. In contrast, the latter frequency band (8–12 Hz) reflects synchronized activity that enhances ankle stiffness. This frequency band has been known to be driven by afferent input and descending input from subcortical areas (Lippold 1970; Grosse and Brown 2003). Moreover, synchronized neuromuscular activity between synergistic muscles at 8–12 Hz, often referred to as “physiological tremor,” generates rhythmic physical oscillations of the joint, which can disrupt precise movement (Mcauley and Marsden 2000; Boonstra et al. 2008b; van der Stouwe et al. 2015; Flood et al. 2019; Laine and Valero-Cuevas 2020). Physiological tremor has been shown to correlate with pupil diameter, a marker of arousal level, indicating that heightened emotional arousal increases physiological tremor (Dirkx et al. 2020, 2023). Therefore, it is possible that emotional arousal modulates synchronized neuromuscular activity between ankle plantarflexors, particularly at 8–12 Hz, during quiet standing. In addition to the above-mentioned frequency bands, weak but significant synchronized activity between the ankle plantarflexors is also observed in the high-frequency range, generally referring to the beta (15–30 Hz) and gamma (30–40 Hz) bands. These high frequency bands have been known to reflect common cortical drive to two different motoneuron pools (Norton and Gorassini 2006; Fisher et al. 2012). Recently, Zaback et al. reported that repeated exposure to postural threat induced paralleled habituation of synchronized ankle plantarflexors activity at 20–40 Hz, which was mainly attributed to beta band modulation, as well as arousal level (Zaback et al. 2022). Cortical contribution during postural threat was further indicated by evidence showing that cortical activity related to COP peaks is more pronounced during postural threat (Zaback et al. 2023). Therefore, synchronized activity between the ankle plantarflexors at 15–30 Hz, which originates from cortical regions, may be enhanced in aroused emotional states.

One way to quantify synchronized neuromuscular activity is by using inter-muscular coherence (IMC), which calculates the correlation between two EMG signals in the frequency domain (Halliday et al. 1995). IMC is commonly used for research exploring neuromuscular mechanisms underlying standing postural control (Obata et al. 2014; Nandi et al. 2019; Nojima et al. 2020). By examining synchronized neuromuscular activity of ankle plantarflexors, we can gain deeper insights into the relationship between emotional states and postural control.

Taken together, the present study aimed to clarify the emotional effects on both individual and synchronized neuromuscular activities of ankle plantarflexors during quiet standing. Based on the report by Horslen and Carpenter (Horslen and Carpenter 2011), we expected that arousal would influence COP variables. In line with this expectation, we hypothesized that TA, SOL, and MG activities would be influenced by arousal (H-1). Additionally, we hypothesized that IMC at 0–4 Hz, 8–12 Hz, and 15–30 Hz between the ankle plantarflexors would also be influenced by arousal (H-2). Finally, we conducted an exploratory investigation into the relationship between changes in COP variables and neuromuscular activities of the ankle plantarflexors using correlation analysis.

## Methods

### Participants

Prior to the experiment, we conducted a power analysis to determine the required number of participants using G*Power (version 3.1). Specifically, we referred to the previous study that reported the effect of arousal on the COP variable (mean power frequency) and used its findings to calculate the sample size (effect size (ηp2): 0.151; α level: 0.05; power (1 − β error probability): 0.95) (Horslen and Carpenter 2011). As a result, the calculated required number of participants was twenty-two. Considering the possibility of data exclusion for some participants, twenty-four healthy male volunteers were recruited for the present study. The mean age, weight, and height of the participants with their respective standard deviations (SD) were 24.5 ± 2.4 years, 66.0 ± 7.2 kg, and 174.3 ± 5.2 cm, respectively. All participants provided written informed consent to participate in the present study, and the experimental procedures were approved by the local ethics committee of the University of Tokyo (Number: 792). This study was performed in accordance with the Declaration of Helsinki (1964). Before the experiment, participants answered 21 questions on the Beck Depression Inventory-II (Beck et al. 1996) to assess their degree of depression. None of the participants exhibited a depression score exceeding 17; consequently, no participants were excluded from the analysis. This cut-off score, which is the criterion for depression, was in line with that used in previous studies (Lebert et al. 2020; Takahashi et al. 2024).

### Affective stimuli and conditions

The International Affective Picture System (IAPS) (Lang et al. 2005) was used for affective stimuli. Self-Assessment Manikin (SAM) (Bradley and Lang 1994) scores were attached to each picture as quantified arousal and valence values. SAM scores ranged from 1 to 9: low values indicated low-arousal or unpleasant-valence, and high values indicated high-arousal or pleasant-valence, respectively. Based on the SAM scores, twelve pictures were selected for the following six conditions comprising two arousal conditions (High and Low) and two valence conditions (Unpleasant and Pleasant): (1) High-Pleasant (Erotic couples and female erotica), (2) High-Unpleasant (Mutilations and dead bodies), (3) Low-Pleasant (Nature, families, and animals), and (4) Low-Unpleasant (Pollution, loss, illness, and contamination). Appendix A. provides the affective pictures used for each condition.

### Data collection

**Fig. 1:**
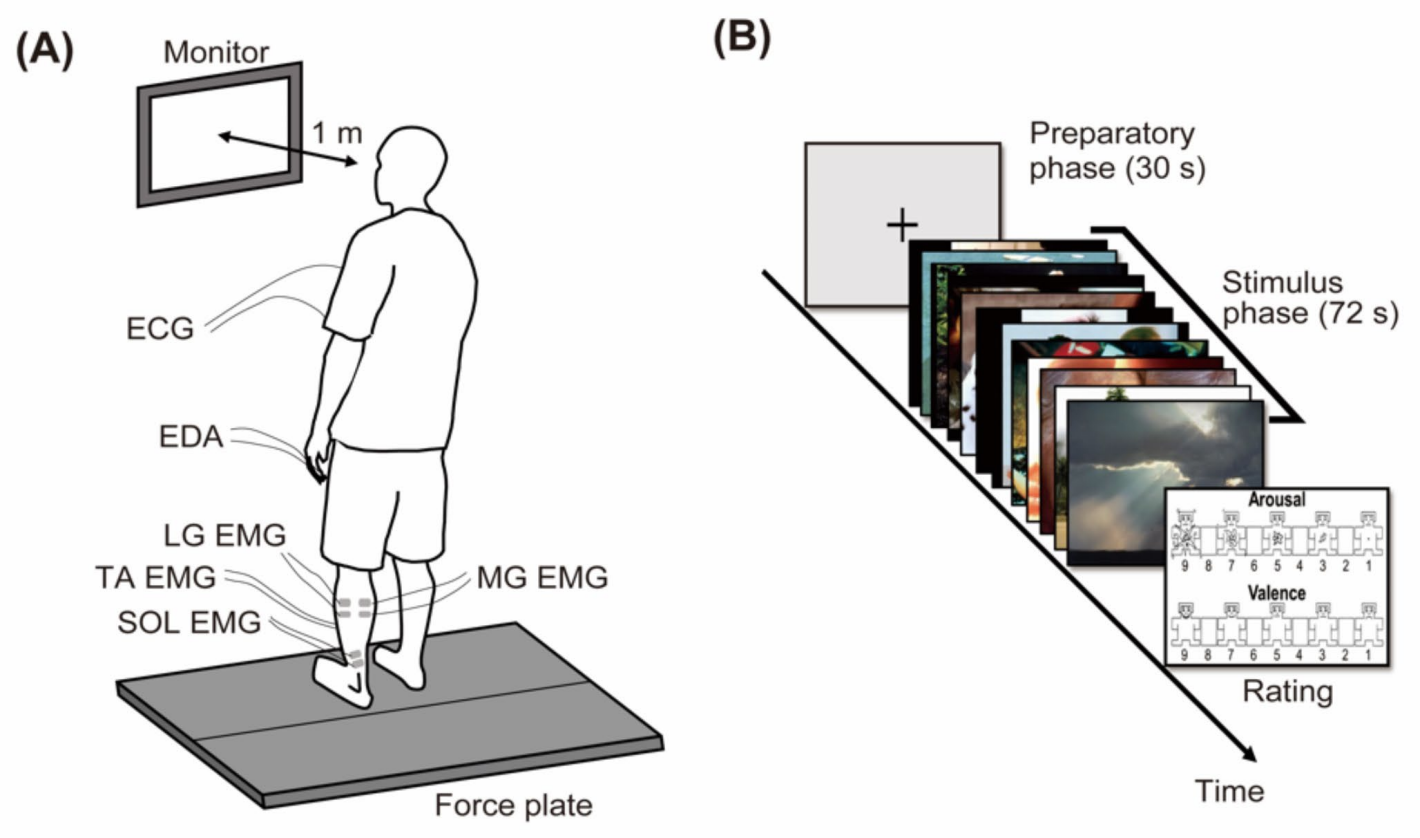
(**A**) Experimental setup. Participants stood on a force plate and looked at a monitor placed 1 m in front of them. Ground reaction forces and moments were recorded using the force plate. Additionally, EMG of TA, SOL, MG, and LG, as well as ECG and EDA were collected using surface electrodes. (**B**) Experimental paradigm for each block. Each block started with a preparatory phase lasting 30 s followed by the stimulus phase lasting 72 s. In the stimulus phase, 12 pictures were displayed for 6 s each. Finally, the participants rated their subjective emotional states using the SAM scores

Figure 1A shows the experimental setup. Ground reaction forces and moments were recorded using a force plate (Bertec, Columbus, OH, USA). EMG, electrocardiogram (ECG), and electrodermal activity (EDA) data were collected using two bipolar Ag/AgCl bipolar surface electrodes (Vitrode F-150 S, Nihon Kohden, Tokyo, Japan). The EMG data were recorded from the tibialis anterior (TA), soleus (SOL), medial head (MG), and lateral head of the gastrocnemius (LG) muscles in the non-dominant leg, following the SENIAM recommendation (Hermens et al. 2000). Two electrodes were placed over the muscle belly with a 1.5 cm separation between the center of electrodes. The ECG data were recorded using a lead II pattern with two electrodes. The EMG and ECG signals were bandpass-filtered (EMG: 5–1000 Hz; ECG: 0.08–1000 Hz) and amplified (×1000) using a multichannel amplifier (MEG-6108, Nihon Kohden, Tokyo, Japan). The EDA data were recorded from two electrodes placed on the middle and ring fingers of the non-dominant hand, and then amplified (×100) using a dedicated unit (AP-U030, Miyuki Giken, Tokyo, Japan). All data were recorded at a sampling rate of 1000 Hz using analog-to-digital (A/D) converters (Ground reaction force/moment, EMG, and ECG: Powerlab/16SP, AD Instruments, Castle Hill, Australia; EDA: MP208, Miyuki Giken, Tokyo, Japan).

### Procedures

Participants were asked to stand on the force plate with their feet shoulder-width apart and their arms relaxed along the trunk (Fig. 1A). A 21.5-inch monitor (S2240Tb, Dell, TX, USA) was placed 1 m in front of the participants, and its height was adjusted to the eye level. The experiment consisted of four blocks, and each block started with a preparatory phase lasting 30 s followed by a stimulus phase lasting 72 s (Fig. 1B). During the preparatory phase, a fixation cross was displayed on the monitor, and then 12 affective pictures for each condition were displayed in each stimulus phase. Each affective picture was displayed successively for 6 s without any interval (6 s × 12 pictures = 72 s). The order of experimental blocks was randomized across participants. A 120-second rest period in a sitting position was set between the blocks. After each block, the participants were asked to rate their subjective arousal and valence scores throughout the block using SAM scores closest to the face of the manikin.

### Data analysis

All signals were analyzed using a custom-written script in MATLAB (2023a, MathWorks Inc., MA, USA). Signals recorded only during the stimulus phase were analyzed because the preparatory phase was intended to stabilize the standing posture and the emotional state. Two participants reported voluntary movements that were not related to spontaneous body sway by themselves. In addition, one participant reported fatigue in the legs during the task. Because these factors can affect postural control, these three participants were excluded from the analysis. Ultimately, 21 participants were included in the analysis.

#### Autonomic nervous activity

EDA data were low-pass filtered at 1 Hz using a second-order Butterworth filter. From the filtered data, the mean amplitude (Mean EDA) was calculated in each block. Because EDA has been shown to be related to sympathetic activity but not parasympathetic activity (Wallin 1981), the sympathetic activity during the task was assessed by the Mean EDA. In addition, the Mean EDA was used as an objective indicator of arousal (Bradley et al. 2001).

All ECG data were low-pass filtered at 20 Hz using a second-order Butterworth filter, and then time-series data of R-R intervals (RRI) were calculated. From the RRI data, the mean heart rate (Mean HR) and the root mean square of successive differences of the RRI (RMSSD) were calculated. The Mean HR is regulated by both sympathetic and parasympathetic (i.e., vagal tone) activities, while the RMSSD reflects parasympathetic activity (Shaffer et al. 2020). Generally, unpleasant emotions have been shown to reduce the HR (i.e., fear bradycardia) regardless of arousal level (Bradley et al. 2001; Gomez and Danuser 2010), which is mainly regulated by an increase in vagal tone (Campbell et al. 1997). Therefore, the RMSSD was used as an objective indicator of valence.

#### COP variables

The force plate signals were low-pass filtered at 15 Hz using a second-order Butterworth filter, and then the COP displacement in the anterior-posterior (AP) and medial-lateral (ML) directions was calculated in each block. To quantify the dynamics of postural control during quiet standing, the following four COP variables were calculated in each direction: (1) mean position (MP-AP and MP-ML), (2) standard deviation (SD-AP and SD-ML), (3) mean velocity (MV-AP and MV-ML), and (4) mean power frequency (MPF-AP and MPF-ML). The MV was determined by dividing the total path length of the COP displacement by the duration of the stimulus phase. For the pre-processing step in calculating the MPF, the DC bias of the COP displacement in each direction was removed by subtracting the mean value from each point. Next, the 72 s data in each block were divided into 12 segments (90% overlapping) with 32,768 data points. A 15-bit fast Fourier transform was applied to each segment after applying a Hanning window to each segment. The frequency resolution was approximately 0.03 Hz (1000 Hz / 32768 data points). Subsequently, the auto-power spectrum density (PSD) of the COP displacement was calculated. Then, mean power frequency (range: 0–15 Hz) was calculated in each direction (MPF-AP and MPF-ML) using Eq. (1):

1 $$\:\text{MPF\:}\text{[Hz]\:=}\text{}\frac{\sum\:f\cdot\:P\left(f\right)}{\sum\:P\left(f\right)}$$

where $\:f$ is the frequency of the COP signal and $\:P$ is the PSD at the frequency.

#### Individual neuromuscular activity

All EMG data were band-pass filtered at 20–450 Hz using a second-order Butterworth filter, and the electrical line noise was removed using a notch-filter at 50 Hz. The filtered EMG signals were full wave rectified. Subsequently, the mean value of the rectified EMG (Mean EMG) for each experimental block was calculated for the TA, SOL, MG, and LG, which was used as an indicator of neuromuscular activity during the task.

Frequency domain analysis was also conducted on the rectified EMG signals. Because the standing posture throughout the task was mainly controlled by the ankle plantarflexors (i.e., SOL, MG, and LG) but not by the ankle dorsiflexor (TA) as in the previous report (Masani et al. 2003), frequency domain analysis was conducted on the ankle plantarflexors. To begin with, the PSDs of the rectified EMGs of the SOL, MG, and LG were calculated. As pre-processing for calculation of the PSD, the 72 s data was divided into 16 segments (50% overlapping) with 8192 data points. A 13-bit fast Fourier transform was applied to each segment after applying a Hanning window to each segment. The frequency resolution was approximately 0.12 Hz (1000 Hz / 8192 data points). Following this, mean values of the PSD (Mean PSD) were calculated at 0–4 Hz, and 4–12 Hz for the SOL, MG, and LG. The former frequency band reflects phasic neuromuscular activity related to postural sway (Masani et al. 2003). In contrast, the latter frequency band has a peak PSD at 8–9 Hz and reflects tonic and isometric neuromuscular activity that enhances ankle stiffness (Winter et al. 1998; Sasagawa et al. 2009b; Boonstra et al. 2015).

#### Inter-muscular coherence (IMC)

Inter-muscular coherence (IMC) using two rectified EMG signals was calculated to quantify the synchronized neuromuscular activity between the plantarflexors. The IMC was calculated for the following pairs: (1) SOL-MG, (2) SOL-LG, and (3) MG-LG. The cross-power spectrum density (CSD) between the two rectified EMG signals was calculated by the fast Fourier transform procedure in the same way as the calculation of the PSDs. Then, the IMC was estimated using Eq. (2):

2 $$\:{\left|{C}_{xy}\left(f\right)\right|}^{2}=\frac{{\left|{S}_{xy}\left(f\right)\right|}^{2}}{{S}_{x}\left(f\right){S}_{y}\left(f\right)}$$

where $\:{S}_{x}\left(f\right)$ and $\:{S}_{y}\left(f\right)$ are the PSDs of $\:x$ and $\:y$, $\:{S}_{xy}\left(f\right)$ is the CSD between $\:x$ and $\:y$. The value of the IMC is in the range from 0 to 1. Zero indicates that neuromuscular activities between two muscles are completely independent within a frequency band, while 1 indicates that they are completely synchronized (Halliday et al. 1995). In addition, we calculated the pooled coherence across the participants to visualize the overall subject trends by conditions. The pooled coherence was estimated using Eq. (3):

3 $$\:{C}_{pooled}={\left|\frac{{\sum\:}_{i=1}^{k}{L}_{i}{C}_{xy}^{i}\left(f\right)}{{\sum\:}_{i=1}^{k}{L}_{i}}\right|}^{2}$$

where $\:{C}_{xy}^{i}\left(f\right)$ is the individual coherence, $\:{L}_{i}$ is the number of segments used to calculate the individual coherence (16), and $\:k$ is the number of the subjects (21). Subsequently, all IMC values were transformed into Fisher’s z-score for statistical analysis (Rosenberg et al. 1998). The upper 95% confidence limit for significant coherence was given by Eq. (4):

4 $$\:CL\:=\:1-{\left(\alpha\:\right)}^{\frac{1}{L-1}}$$

where $\:\alpha\:$ is the significance level (0.05) and $\:L$ is the number of segments to calculate the coherence (Halliday et al. 1995). Subsequently, mean values of the IMC (Mean IMC) were calculated at 0–4, 8–12 Hz, 15–30 Hz, and 30–40 Hz, for each muscle pair, regardless of the confidence limit (Obata et al. 2014; Nandi et al. 2019; Nojima et al. 2020). The IMC at 0–4 Hz is related to postural sway (Masani et al. 2003), the IMC at 8–12 Hz corresponds to physiological tremor and is related to the enhancement of ankle stiffness which is driven from afferent and subcortical inputs (Lippold 1970; Grosse and Brown 2003; Kouzaki and Fukunaga 2008), and the IMC at 15–30 and 30–40 Hz reflects cortical drive to muscles (Norton and Gorassini 2006; Fisher et al. 2012).

### Statistical analysis

All statistical comparisons were performed using the R software package (ver. 4.1.2).

#### Comparison among conditions

Shapiro-Wilk tests were conducted to confirm the normal distribution of the variables, and most variables showed non-normal distribution. In line with our previous work (Takahashi et al. 2024), all variables were transformed using the aligned rank transformation procedure in the R package “ARTool” (Kay et al. 2021). The aligned rank transformation allows non-normally distributed data to be applied to the analysis of variance (ANOVA) of the parametric method (Wobbrock et al. 2011). Two-way repeated measures ANOVA with arousal (High and Low) and valence (Unpleasant and Pleasant) as within-subject factors was then performed on the measured variables. A post-hoc contrast test in ARTool was conducted when a significant interaction was observed (Elkin et al. 2021), and p-*values* were subsequently corrected using the false discovery rate (FDR) correction, following the Benjamini-Hochberg procedure. The effect sizes for the ANOVA and post-hoc tests were calculated as partial eta squared (*η*_*p*_^*2*^) and Cohen’s d (*d*), respectively. The significance level for all tests was set to *p* < 0.05.

#### Correlation analysis

We examined whether COP changes was linked to specific modulation of neuromuscular activity of ankle plantarflexors induced by emotional stimuli using correlation analysis. If the two-way ANOVA showed a significant main effect of arousal on the COP and EMG variables, the change ratio was calculated using Eq. (5):

5 $$\:\text{C}\text{h}\text{a}\text{n}\text{g}\text{e}\:\text{r}\text{a}\text{t}\text{i}\text{o}\:=\:\frac{{M}_{High}-{M}_{Low}}{{M}_{Low}}\:$$

Where $\:{M}_{High}$ is the value in High-Arousal and $\:{M}_{Low}$ is the value in Low-Arousal. The change ratio for MP-AP and MP-ML was calculated only by subtracting the values in Low arousal from those in High arousal (i.e., $\:{M}_{High}-{M}_{Low})$. In contrast, if the two-way ANOVA showed a significant main effect of valence on the COP and EMG variables, the change ratio was calculated using Eq. (6):

6 $$\:\text{C}\text{h}\text{a}\text{n}\text{g}\text{e}\:\text{r}\text{a}\text{t}\text{i}\text{o}\:=\:\frac{{M}_{Pleasant}-{M}_{Unpleasant}}{{M}_{Unpleasant}}\:$$

Where $\:{M}_{Pleasant}$ is the value in Pleasant and $\:{M}_{Unpleasant}$ is the value in Unpleasant. The change ratio for MP-AP and MP-ML was calculated only by subtracting the values in Unpleasant from those in Pleasant (i.e., $\:{M}_{Pleasant}-{M}_{Unpleasant})$. Then, we calculated Kendall’s correlation coefficient (*τ*) between the change ratio of the COP and EMG variables. All *p-values* calculated in the correlation analysis were uncorrected, and the significance level for all tests was set to *p* < 0.05.

## Results

### Subjective emotional ratings

**Fig. 2:**
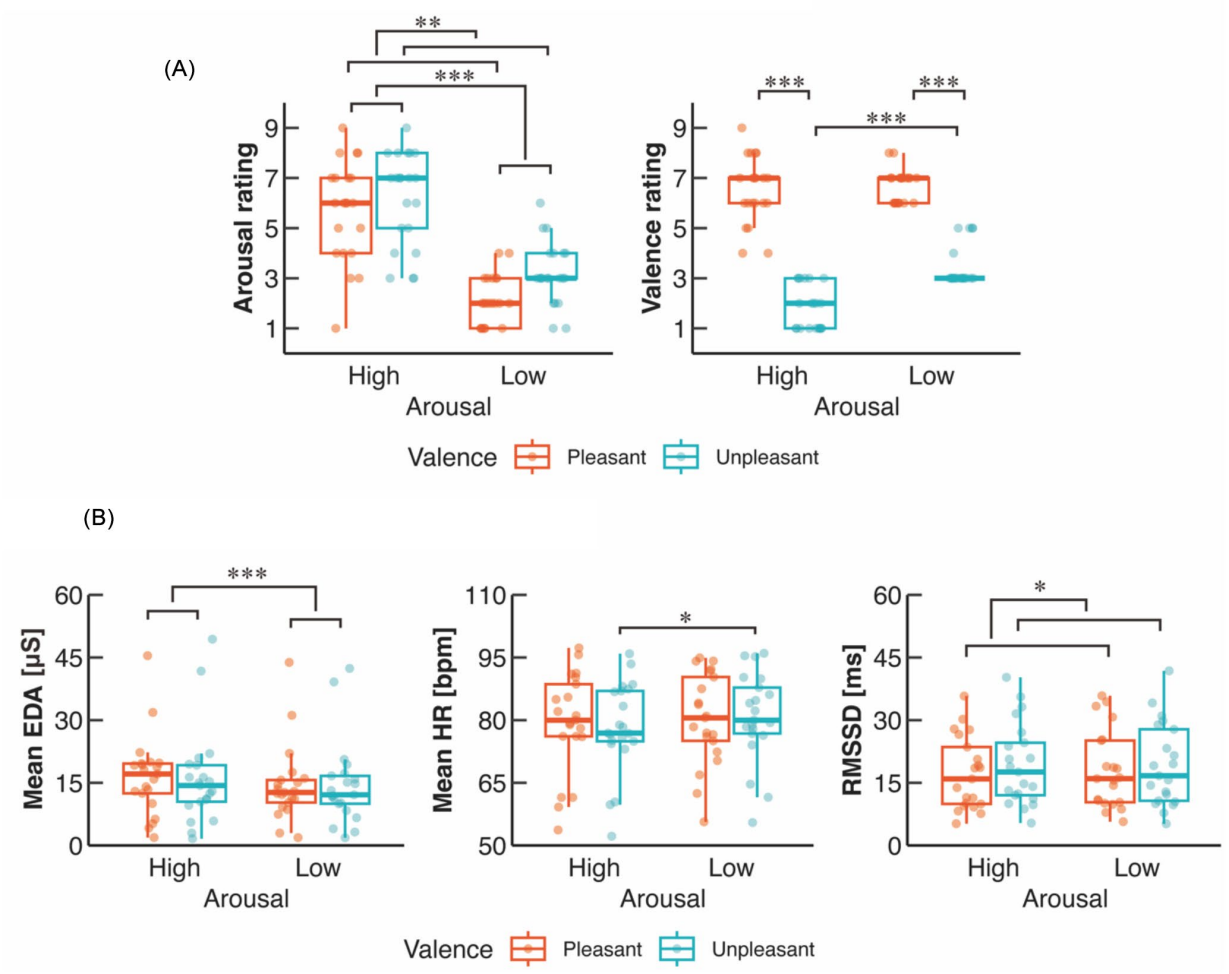
Group data for (**A**) subjective emotional ratings and (**B**) autonomic nervous activity. The lines in the box plots indicate median values, and the ends of the boxes represent the 25th and 75th percentiles. The dots represent individual data points. *p < 0.05, ***p < 0.001

Table 1 summarizes the statistic, *p*-values, and effect sizes related to two-way ART-ANOVA. The two-way ART-ANOVA (two arousal × two valence) revealed significant main effects of arousal (*p* < 0.001) and valence (*p* = 0.00396) on the arousal rating (Table 1; Fig. 2A). The arousal rating was significantly higher in High arousal than in Low arousal, and higher in Unpleasant than in Pleasant. We did not observe a significant interaction on the arousal rating (*p* = 0.253). Moreover, the two-way ART-ANOVA showed significant main effects of arousal (*p* < 0.001) and valence (*p* < 0.001) as well as an interaction (*p* = 0.00227) on the valence rating. Post-hoc contrast tests showed that the valence rating was significantly lower (lower pleasantness) in High-Unpleasant than in High-Pleasant (*p*_*FDR*_ < 0.001) and Low-Unpleasant (*p*_*FDR*_ < 0.001). In addition, the valence rating was significantly lower in Low-Unpleasant than in Low-Pleasant (*p*_*FDR*_ < 0.001). The valence rating in High-Pleasant was not significantly different from that in Low-Pleasant (*p*_*FDR*_ = 0.649).

**Table 1:** Summary of the two-way ART-ANOVA (arousal × valence) of all variables. Significant *p*-values (< 0.05) are in boldface

	Arousal main effect				Valence main effect				Arousal × Valence interaction		
	*F*_(1, 60)_	*p-value*	*η*_*p*_^*2*^		*F*_(1, 60)_	*p-value*	*η*_*p*_^*2*^		*F*_(1, 60)_	*p-value*	*η*_*p*_^*2*^
*Subjective emotional ratings*											
Arousal rating	158	**< 0.001**	0.724		8.98	**0.00396**	0.130		1.33	0.253	0.022
Valence rating	15.8	**< 0.001**	0.208		251	**< 0.001**	0.807		10.2	**0.00227**	0.145
*Autonomic nervous activity*											
Mean EDA	13.4	**< 0.001**	0.183		2.56	0.114	0.041		2.62	0.110	0.042
Mean HR	4.09	**0.0475**	0.064		1.05	0.310	0.017		4.79	**0.0326**	0.074
RMSSD	< 0.001	0.982	< 0.001		5.24	**0.0256**	0.080		0.312	0.578	0.005
*COP variables*											
MP-AP	3.08	0.0845	0.049		0.631	0.431	0.010		0.459	0.500	0.008
SD-AP	0.726	0.397	0.011		1.17	0.283	0.019		0.011	0.920	< 0.001
MV-AP	1.99	0.163	0.032		0.206	0.651	0.003		0.005	0.942	< 0.001
MPF-AP	14.1	**< 0.001**	0.190		0.503	0.480	0.008		0.868	0.355	0.014
MP-ML	2.38	0.128	0.038		0.404	0.527	0.007		0.209	0.649	0.003
SD-ML	0.579	0.449	0.010		2.48	0.120	0.039		2.04	0.158	0.033
MV-ML	0.598	0.442	0.010		0.317	0.575	0.005		0.343	0.560	0.006
MPF-ML	3.02	0.0873	0.048		0.049	0.826	0.001		1.95	0.167	0.032
*Individual muscle activity*											
***Mean EMG***											
TA	0.217	0.643	0.004		0.029	0.864	< 0.001		0.322	0.572	0.005
SOL	5.14	**0.0270**	0.079		0.880	0.351	0.014		1.02	0.316	0.017
MG	2.14	0.148	0.034		1.09	0.301	0.018		0.800	0.374	0.013
LG	3.03	0.0870	0.048		0.139	0.710	0.002		0.552	0.460	0.009
***Mean PSD***											
SOL (0–4 Hz)	4.29	**0.0427**	0.067		0.003	0.953	0.000		1.02	0.315	0.017
MG (0–4 Hz)	0.037	0.847	0.001		0.108	0.743	0.002		2.20	0.143	0.035
LG (0–4 Hz)	3.59	0.0631	0.056		0.078	0.781	0.001		0.920	0.781	0.001
SOL (4–12 Hz)	4.52	**0.0376**	0.070		0.282	0.597	0.005		0.678	0.413	0.011
MG (4–12 Hz)	0.030	0.862	0.001		2.12	0.150	0.034		0.421	0.518	0.007
LG (4–12 Hz)	1.41	0.240	0.023		0.084	0.772	0.001		0.567	0.454	0.009
*Inter-muscular coherence*											
***Mean IMC***											
SOL-MG (0–4 Hz)	0.87	0.353	0.014		0.80	0.374	0.013		0.13	0.723	0.002
SOL-LG (0–4 Hz)	1.26	0.265	0.021		0.283	0.596	0.005		0.430	0.514	0.007
MG-LG (0–4 Hz)	0.619	0.434	0.010		3.887	0.0532	0.061		0.06	0.801	0.001
SOL-MG (8–12 Hz)	4.56	**0.0368**	0.071		1.08	0.303	0.018		0.03	0.874	< 0.001
SOL-LG (8–12 Hz)	0.274	0.602	0.005		0.014	0.905	< 0.001		0.603	0.440	0.010
MG-LG (8–12 Hz)	4.00	**0.0499**	0.063		0.67	0.416	0.011		0.038	0.845	0.001
SOL-MG (15–30 Hz)	4.05	**0.0485**	0.063		0.571	0.452	0.009		0.473	0.494	0.008
SOL-LG (15–30 Hz)	5.20	**0.0261**	0.080		1.38	0.245	0.022		2.24	0.140	0.036
MG-LG (15–30 Hz)	0.617	0.435	0.010		3.78	0.0565	0.059		0.644	0.425	0.011
SOL-MG (30–40 Hz)	0.031	0.860	< 0.001		0.676	0.414	0.011		1.14	0.290	0.019
SOL-LG (30–40 Hz)	4.92	**0.0304**	0.076		0.023	0.879	< 0.001		0.747	0.390	0.012
MG-LG (30–40 Hz)	1.49	0.227	0.024		0.342	0.561	0.006		0.061	0.805	0.001

### Autonomic nervous activity

The two-way ART-ANOVA showed a significant main effect of arousal (*p* < 0.001) on the mean EDA (Table 1; Fig. 2B). The Mean EDA was significantly higher in High arousal than in Low arousal. We did not find a significant main effect of valence (*p* = 0.114) or a significant interaction (*p* = 0.110) on the Mean EDA.

As for the ECG data, the two-way ART-ANOVA showed significant main effect of arousal (*p* = 0.0475) and interaction (*p* = 0.0326) but not a main effect of valence (*p* = 0.310) on the Mean HR (Table 1; Fig. 2B). The post-hoc tests revealed that the Mean HR was significantly lower in High-Unpleasant than in Low-Unpleasant (*p*_*FDR*_ = 0.0128, *d* = 0.307). In contrast, the Mean HR in High-Unpleasant was not significantly different from that in High-Pleasant (*p*_*FDR*_ = 0.0520, *d* = 0.251). The Mean HR in Low-Pleasant was not significantly different from those in High-Pleasant (*p*_*FDR*_ = 0.923, *d* = 0.019) and Low-Unpleasant (*p*_*FDR*_ = 0.377, *d* = 0.096). In addition, the two-way ART-ANOVA showed a significant main effect of valence on the RMSSD (*p* = 0.0256), indicating a greater value in Unpleasant than in Pleasant. These results demonstrate that manipulations of arousal and valence were successful.

### COP variables

**Fig. 3:**
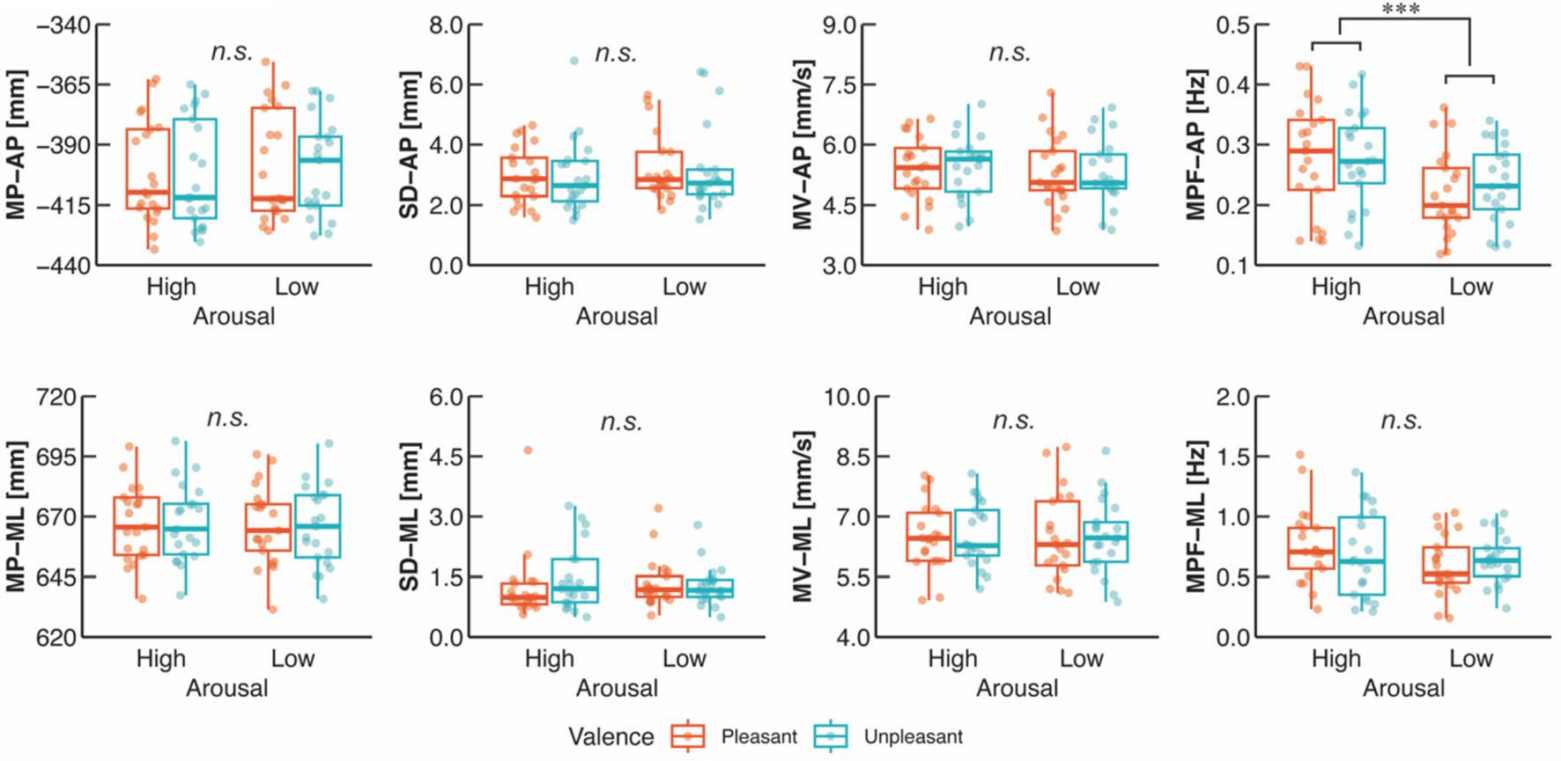
Group data for COP variables. For the MP-AP and MP-ML, larger values indicate a forward lean and a right lean, respectively. The lines in the box plots indicate median values, and the ends of the boxes represent the 25th and 75th percentiles. The dots represent individual data points. *n.s.*, non-significant; ***p < 0.001

The two-way ART-ANOVA showed a significant main effect of arousal (*p* < 0.001) but no significant main effect of valence (*p* = 0.480) or interaction (*p* = 0.355) on the MPF-AP (Table 1; Fig. 3). The MPF-AP was significantly higher in High arousal than Low arousal. For the other COP variables, any significant main effect or interaction was not observed (All *p* > 0.05).

### Individual neuromuscular activity

**Fig. 4:**
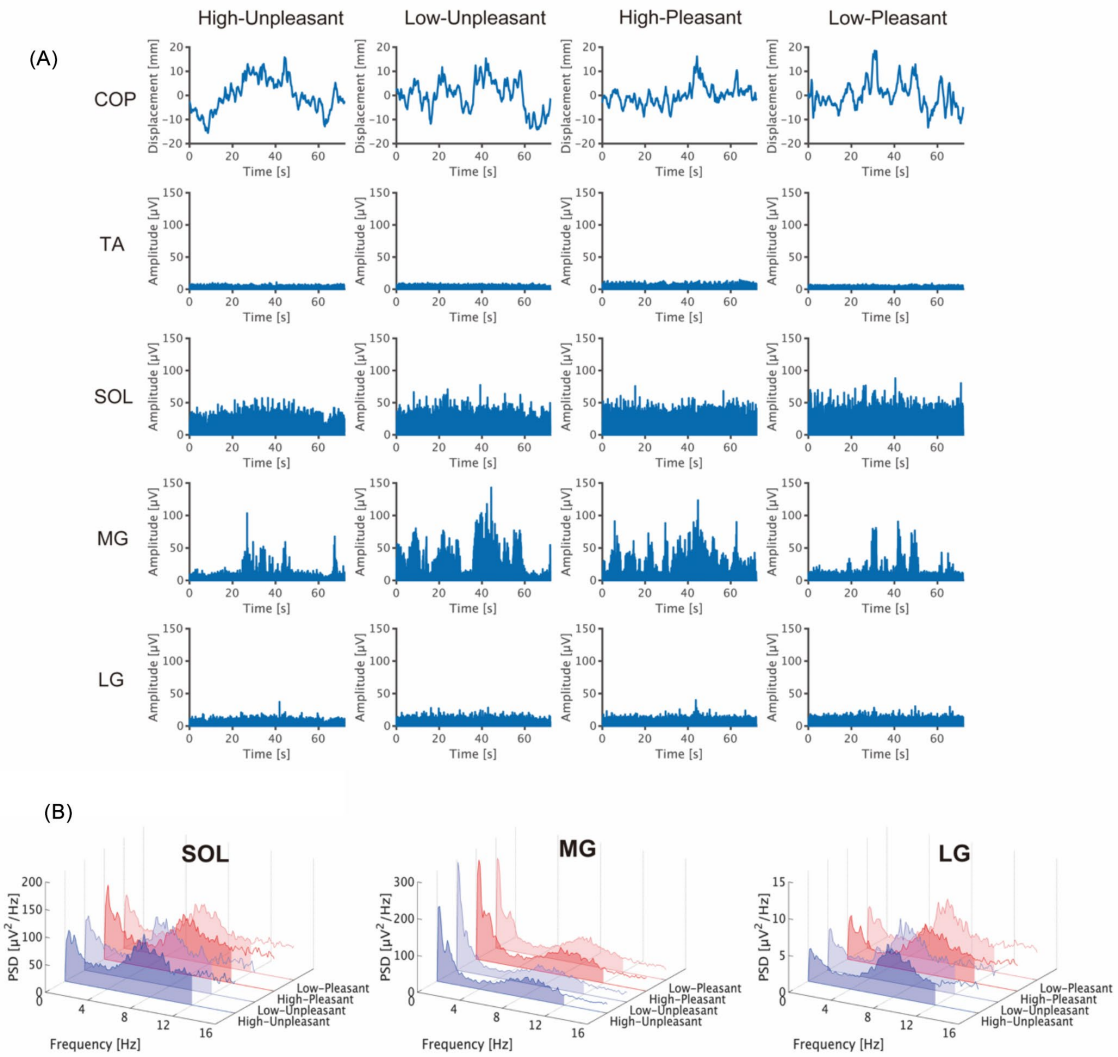
(**A**) Representative waveform from a participant. Each graph shows the COP displacement in the AP direction and the rectified EMG for the stimulus phase in each experimental block. In the vertical axis of the COP displacement, positive values correspond to the anterior direction, while negative values correspond to the posterior direction. (**B**) Pooled PSD of EMG across participants. The frequency band to be analyzed is highlighted in filled areas

**Fig. 5:**
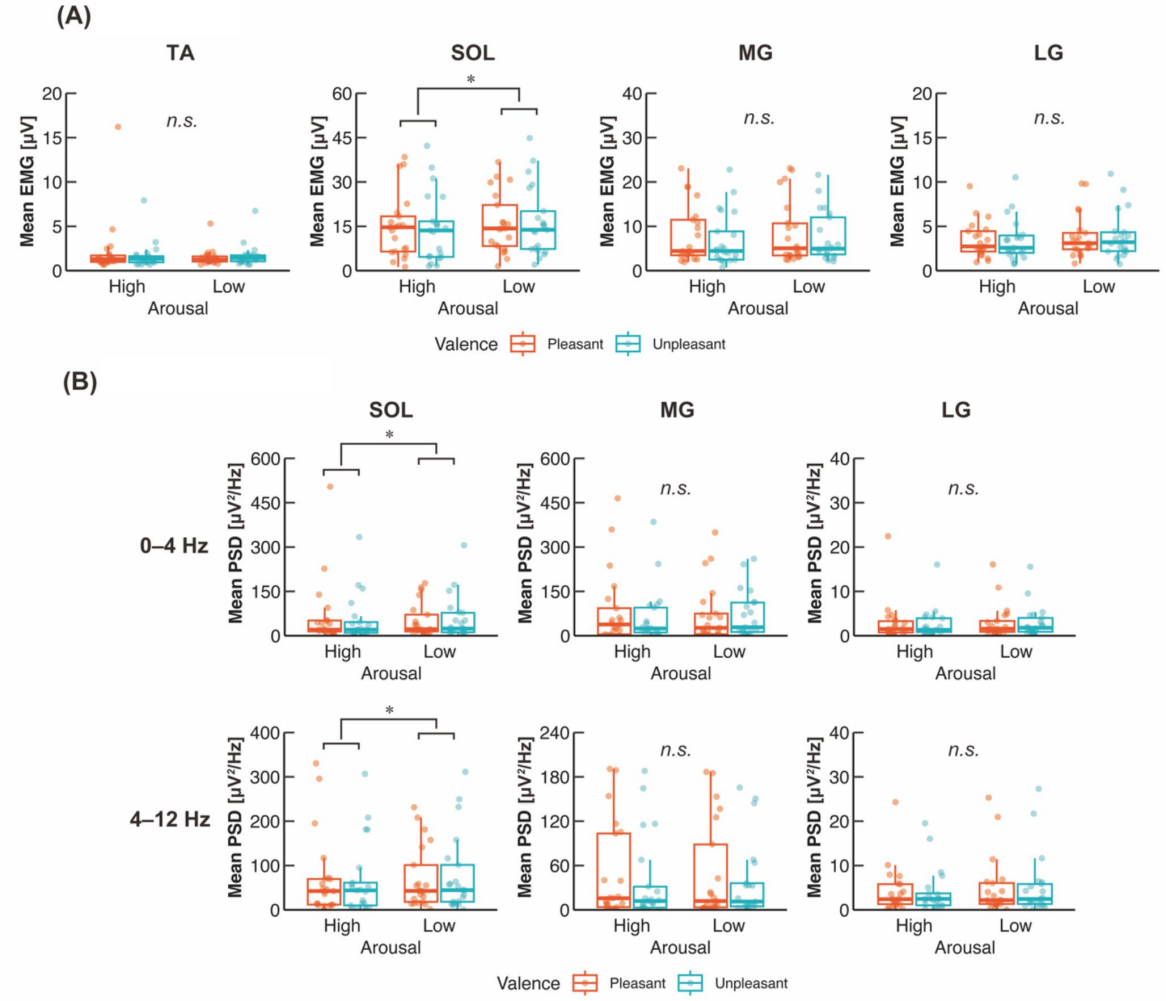
Group data for (**A**) Mean EMG and (**B**) Mean PSD of EMG. The lines in the box plots indicate median values, and the ends of the boxes represent the 25th and 75th percentiles. The dots represent individual data points. *n.s.*, non-significant; *p < 0.05

Figure 4A shows representative COP displacement in the AP direction and rectified EMG data for each muscle and condition. Ankle dorsiflexor (i.e., TA) activity seems to be absent compared with ankle plantarflexor activities (i.e., SOL, MG, and LG). In addition, while the SOL and LG show tonic and constant activation, the MG exhibits phasic and intermittent activation corresponding to the COP sway. These activation patterns of ankle dorsi/plantarflexor in young participants are consistent with a previous report (Masani et al. 2003). Regarding the mean neuromuscular activity in each block, the two-way ART-ANOVA showed a significant main effect of arousal (*p* = 0.0270) but no significant main effect of valence (*p* = 0.351) or interaction (*p* = 0.316) on the Mean EMG of the SOL (Table 1; Fig. 5A). The Mean EMG of the SOL was lower in High arousal than in Low arousal. The Mean EMGs of the TA, MG, and LG were not significantly affected by arousal, valence, or their interaction (All *p* > 0.05).

Figure 4B shows pooled PSDs across the participants for each muscle and condition. Two major components were found at 0–4 Hz, and 4–12 Hz with a peak at around 8 Hz, for each muscle and condition. The two-way ART-ANOVA showed significant main effects of arousal but no significant main effect of valence or interaction on the Mean PSDs at 0–4 Hz (*p* = 0.0427) and 4–12 Hz (*p* = 0.0376) of the SOL (Table 1; Fig. 5B). The Mean PSDs at 0–4 and 4–12 Hz of the SOL were significantly lower in High arousal than in Low arousal. In contrast, no significant main effect or interaction was observed at either frequency band of the MG or LG (All *p* > 0.05).

### Inter-muscular coherence

**Fig. 6:**
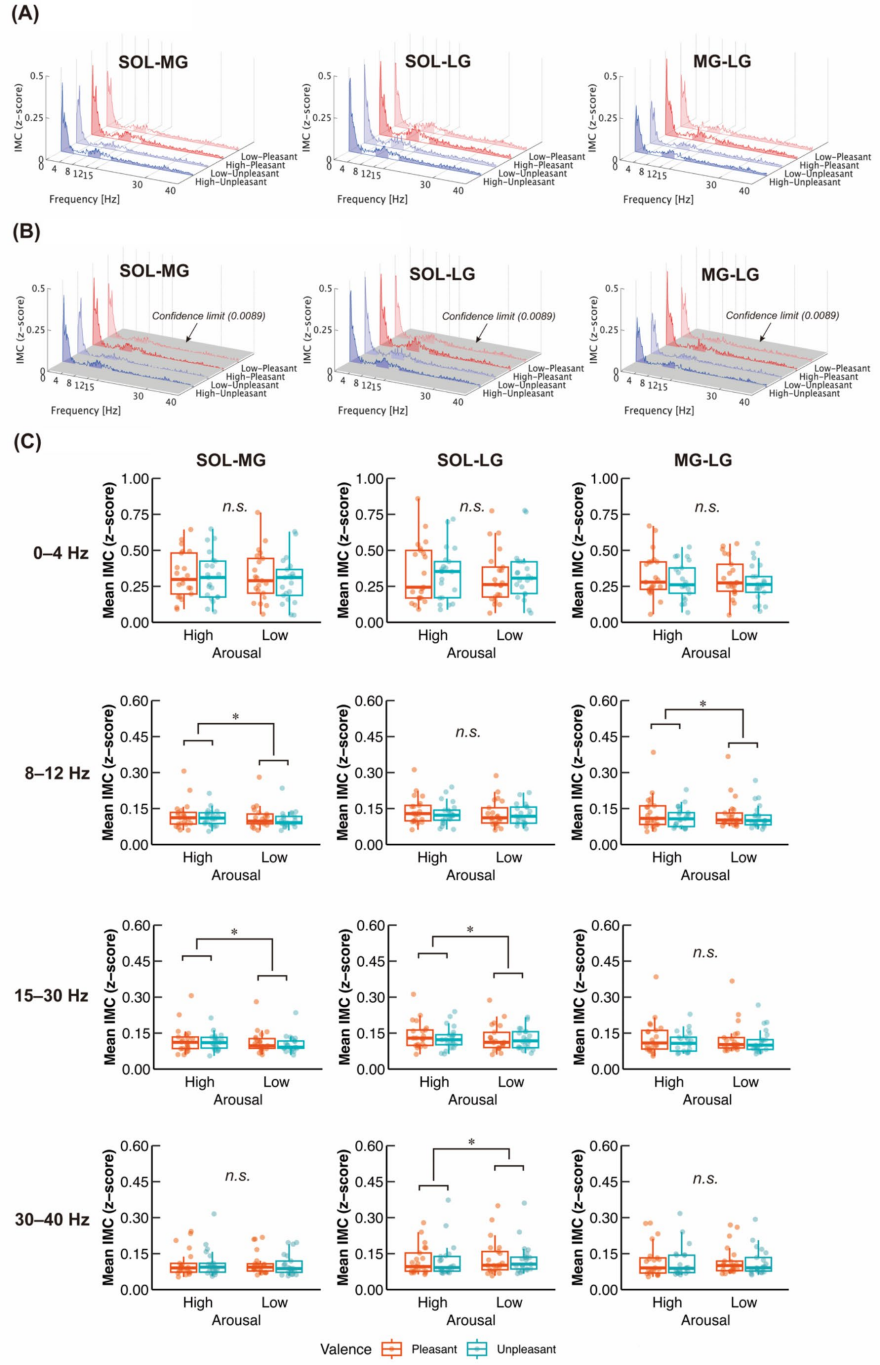
(**A**) Pooled IMC across participants. The frequency band analyzed is highlighted in filled areas. (**B**) Pooled IMC with confidence limit across participants. Gray plane indicates the confidence limit value (0.0089). (**C**) Group data for Mean IMC transformed to z-score. The lines in the box plots indicate median values, and the ends of the boxes represent the 25th and 75th percentiles. The dots represent individual data points. *n.s.*, non-significant; *p < 0.05

Figure 6A and B shows pooled IMCs across the participants for each muscle pair and condition. A major component was found at 0–4 Hz for each muscle pair and condition. Additionally, a non-negligible peak was found at around 10 Hz. Significant coherences above the confidence limit (0.0089) were distributed up to 40 Hz (Fig. 6B). Regarding 0–4 Hz, we did not find any significant main effect or interaction on the Mean IMC of all muscle pairs (All *p* > 0.05) (Table 1; Fig. 6C). For 8–12 Hz, we found significant main effects of arousal on the Mean IMC of SOL-MG (*p* = 0.0368) and MG-LG (*p* = 0.0499), showing greater IMC values in aroused emotions, but not SOL-LG (*p* = 0.602). For 15–30 Hz, significant main effects of arousal were observed in the Mean IMC of SOL-MG (*p* = 0.0485) and SOL-LG (*p* = 0.0261) with increased IMC values in aroused emotions, but not in MG-LG (*p* = 0.435). Regarding 30–40 Hz, significant main effect of arousal was observed in the Mean IMC of SOL-LG (*p* = 0.0304) with decreased IMC value in aroused emotions, but not in SOL-MG (*p* = 0.860) or MG-LG (*p* = 0.227).

### Correlation analysis

Since the two-way ART-ANOVA showed significant main effect of arousal but not valence on some COP and EMG variables, we calculated the change ratio of each variable using Eq. (5). Kendall’s correlation coefficient revealed that MP-AP was significantly correlated with Mean EMG of SOL (*τ* = 0.371; *p* = 0.0186), Mean EMG of MG (*τ* = 0.495; *p* = 0.00130), Mean EMG of LG (*τ* = 0.419; *p* = 0.00743), Mean PSD of SOL at 4–12 Hz (*τ* = 0.333; *p* = 0.0360), Mean PSD of MG at 4–12 Hz (*τ* = 0.410; *p* = 0.00902), Mean PSD of LG at 4–12 Hz (*τ* = 0.438; *p* = 0.00497), and Mean IMC of SOL-MG at 0–4 Hz (*τ* = −0.390; *p* = 0.0131) (Table 2). SD-AP and Mean PSD of SOL at 0–4 Hz (*τ* = 0.324; *p* = 0.0419) were also significantly correlated. MV-AP was significantly correlated with Mean IMC of SOL-LG at 15–30 Hz (*τ* = 0.324; *p* = 0.0419). In addition, MPF-ML was significantly correlated with Mean EMG of MG (*τ* = −0.352; *p* = 0.0261), Mean EMG of LG (*τ* = −0.314; *p* = 0.0487), and Mean PSD of MG at 4–12 Hz (*τ* = −0.362; *p* = 0.0221). SD-ML was significantly correlated with Mean IMC of SOL-LG at 30–40 Hz (*τ* = 0.371; *p* = 0.0186).

**Table 2:** Kendall’s correlation coefficient (*τ*) between COP variables and EMG variables. Correlation coefficients with significant *p*-values are in boldface. *p < 0.05; **p < 0.01

	COP variables							
	MP-AP	SD-AP	MV-AP	MPF-AP	MP-ML	SD-ML	MV-ML	MPF-ML
***Mean EMG***								
TA	0.171	0.219	0.267	−0.076	−0.133	0.276	0.124	−0.162
SOL	0.371*	0.133	0.105	−0.181	0.143	0.076	−0.210	−0.057
MG	0.495**	0.067	0.076	−0.190	0.133	0.219	−0.143	−0.352*
LG	0.419**	0.124	0.076	−0.152	0.229	0.162	−0.124	−0.314*
***Mean PSD***								
SOL (0–4 Hz)	0.200	0.324*	0.276	−0.105	−0.124	0.000	−0.057	0.000
MG (0–4 Hz)	0.171	0.162	0.286	0.076	−0.114	0.124	−0.086	−0.238
LG (0–4 Hz)	0.324*	0.257	0.190	−0.133	0.057	0.181	−0.086	−0.238
SOL (4–12 Hz)	0.333*	0.133	0.143	−0.067	0.029	−0.019	−0.095	0.019
MG (4–12 Hz)	0.410**	0.133	0.124	−0.143	0.029	0.229	−0.114	−0.362*
LG (4–12 Hz)	0.438**	0.162	0.038	−0.152	0.152	0.219	−0.181	−0.238
***Mean IMC***								
SOL-MG (0–4 Hz)	−0.390*	−0.057	0.048	0.200	−0.124	−0.267	0.095	0.229
SOL-LG (0–4 Hz)	−0.219	0.114	0.238	0.257	−0.124	−0.210	0.267	0.190
MG-LG (0–4 Hz)	−0.086	0.114	0.143	0.219	0.010	−0.152	0.133	0.190
SOL-MG (8–12 Hz)	0.010	−0.171	−0.124	−0.010	0.067	−0.133	−0.019	0.095
SOL-LG (8–12 Hz)	−0.114	−0.143	−0.076	0.152	−0.21	−0.143	−0.181	0.219
MG-LG (8–12 Hz)	−0.038	−0.238	−0.229	−0.095	0.019	−0.029	−0.181	0.086
SOL-MG (15–30 Hz)	−0.076	−0.029	0.057	0	−0.076	−0.162	−0.067	0.181
SOL-LG (15–30 Hz)	−0.133	0.181	0.324*	0.076	−0.324	−0.086	0.048	0.181
MG-LG (15–30 Hz)	−0.076	0.086	0.190	−0.133	−0.114	−0.048	−0.010	0.086
SOL-MG (30–40 Hz)	0.257	0.076	0.143	0.029	−0.067	0.133	0.019	−0.095
SOL-LG (30–40 Hz)	0.305	0.162	0.248	0.000	−0.038	0.371*	0.029	−0.257
MG-LG (30–40 Hz)	0.181	0.095	−0.124	-0.181	0.048	−0.114	−0.190	0.114

## Discussion

The present study aimed to clarify the emotional effects on both individual and synchronized neuromuscular activities of ankle plantarflexors during quiet standing. Our findings first demonstrated that sympathetic activity was affected by arousal, while parasympathetic activity (i.e., vagal tone) was influenced by valence (Fig. 2B). These results demonstrate that the manipulations of arousal and valence were successful. Regarding the COP variables, aroused emotions increased MPF-AP irrespective of valence level (Fig. 3), consistent with the previous finding (Horslen and Carpenter 2011). Additionally, we found that aroused emotions reduced neuromuscular activity of SOL but not TA, MG, or LG (Fig. 5A; B), which partially supported our initial hypothesis (H-1). However, partially supporting hypothesis H-2, aroused emotions increased synchronized neuromuscular activity at 8–12 and 15–30 Hz, while it reduced synchronized neuromuscular activity at 30–40 Hz between specific parts of ankle plantarflexors (Fig. 6B). Synchronized neuromuscular activity at 0–4 Hz was not significantly influenced by emotions. Additionally, we found significant correlations between COP variables and neuromuscular activities of the ankle plantarflexors. Below, we discuss the detailed mechanisms behind these findings.

### Reduction of neuromuscular activity of SOL in aroused emotions

The present study revealed a reduction in the neuromuscular activity of SOL in aroused emotions, which was significantly associated with certain postural behaviors (i.e., MP-AP and SD-AP) (Table 2). First, Mean PSD of SOL at 0–4 Hz was significantly positively correlated with SD-AP. This finding is consistent with previous studies that reported relationship between the 0–4 Hz EMG component of ankle plantarflexors and postural sway (Masani et al. 2003; Sasagawa et al. 2009b). Second, Mean PSD of SOL at 4–12 Hz was significantly positively correlated with MP-AP. Since MP-AP reflects the average value of the ankle torque, it is reasonable to assume that the 4–12 Hz EMG component, which represents the tonic neuromuscular activity required to maintain constant ankle torque, would be associated with MP-AP from the functional perspective (Sasagawa et al. 2009b). Additionally, aroused emotions also showed a trend toward backward COP leaning (*p* = 0.0845) (Table 1; Fig. 3). Integrating these results suggests that aroused emotions reduce the tonic neuromuscular activity of SOL, potentially leading to partial backward COP leaning (i.e., backward body leaning). Similar findings have been observed when participants experienced fear and anxiety related to postural balance (i.e., postural threat) at height. Specifically, the postural threat led to aroused emotions, a reduction in SOL activity, and backward COP leaning (Carpenter et al. 2001a; Zaback et al. 2019, 2021). However, the underlying postural mechanism differs between postural threat conditions and the picture-elicited emotional condition in the present study. While postural threat involves both conscious stabilization efforts and emotional modulation, the picture-elicited emotional condition primarily elicits emotional modulation (Huffman et al. 2009; Zaback et al. 2016). Despite these differences, the above-mentioned reports on postural threat studies support the current findings, as aroused emotions are present in both scenarios.

Although the neuromuscular activity of SOL was reduced in aroused emotions, no significant changes were observed in MG or LG (Table 1; Fig. 5). Based on Henneman’s size principle, the recruitment threshold of motor units is lower in SOL than in MG and LG during quiet standing (Héroux et al. 2014). Because body sway during quiet standing is minimal, requiring only a small amount of torque to return the body to the equilibrium point, SOL can produce most of the ankle torque sufficient for maintaining postural balance (Masani et al. 2008). Therefore, SOL activity is more tonic and pronounced compared to MG and LG during quiet standing, which can make SOL more sensitive to emotional factors than MG and LG. From an anatomical perspective, the physiological cross-sectional area of SOL is twice as large as the combined total of MG and LG (Yamaguchi et al. 1990). This also supports the critical role of SOL in producing ankle torque during quiet standing. Taken together, selective neural modulation of SOL appears logical from the perspective of ankle torque adjustment.

In addition, TA activity was absent across all conditions and was not significantly influenced by emotional factors (Fig. 6). Originally, we expected that TA activity would have been increased under aroused emotions, which comes from the reports that TA activity is increased under postural threat (Adkin and Carpenter 2018). However, given that postural threat influences not only arousal level but also psychological and attentional states specific to postural threat (Zaback et al. 2019, 2021), the latter factors might be sensitive to TA activity. Typically, quiet standing in young individuals is primarily controlled by the ankle plantarflexors, with minimal or no activation of the ankle dorsiflexors (Masani et al. 2003). On the other hand, TA activation can induce co-contraction of ankle dorsiflexor and plantaflexors, which is energetically inefficient for postural control (Morasso and Sanguineti 2002). Collectively, additional TA activity is not required in aroused emotions, indicating that postural control is managed solely by the ankle plantarflexors, thereby maintaining energetically efficient control.

### Enhancement of synchronized neuromuscular activity at 8–12 Hz between ankle plantarflexors in aroused emotions

The IMC results showed that aroused emotions increased synchronized neuromuscular activity between specific pairs of ankle plantarflexors (SOL-MG and MG-LG) at 8–12 Hz (Fig. 6C). Synchronized neuromuscular activity between synergist muscles at 8–12 Hz is a basic mechanism underlying physiological tremor, which generates rhythmic physical oscillation of the joint (McAuley and Marsden 2000; Boonstra et al. 2008b; van der Stouwe et al. 2015; Flood et al. 2019; Laine and Valero-Cuevas 2020). Therefore, the current findings would reflect the enhanced physiological tremor during aroused emotional states, regardless of valence. This aligns with previous research showing that pupil diameter, which reflects arousal levels, was associated with physiological tremor (Dirkx et al. 2020, 2023).

EMG signals at 8–12 Hz have been suggested to reflect two mechanisms: (1) afferent input from the peripheral nerve (Lippold 1970; Young and Hagbarth 1980; Christakos et al. 2006; Laine et al. 2016) and (2) descending input from subcortical areas (Grosse and Brown 2003) to the spinal motoneuron pool. Regarding the afferent input, it is possible that aroused emotions increased stretch reflex excitability. In a previous study, sympathetic activation enhanced stretch reflex excitability but not H-reflex excitability of SOL in a sitting posture, suggesting that sympathetic activation selectively increased muscle spindle sensitivity (Kamibayashi et al. 2009). Similarly, increased stretch reflex excitability of SOL, but not H-reflex excitability, was reported when sympathetic activity was increased under the postural threat during quiet standing (Horslen et al. 2013). The increased sensitivity of the muscle spindle due to sympathetic activation may be linked to direct neural input from the sympathetic nerve to the muscle spindle (Barker and Saito 1981; Radovanovic et al. 2015) and the facilitation of gamma motoneuron excitability (Kamibayashi et al. 2009). Given that our study showed sympathetic activation in response to aroused emotions (Fig. 2B), it is plausible that aroused emotions facilitated common neural input to the muscle spindles of the ankle plantarflexors. Regarding the descending input from subcortical areas, several neural origins could be involved. When a loud auditory stimulus induced a startle reflex mediated through the reticulospinal tract, a significant IMC between upper limb muscles was observed with a peak at around 12 Hz, suggesting that the IMC at the alpha-band (8–12 Hz) partially reflects the reticulospinal tract (Grosse and Brown 2003). Also, the reticulospinal tract partially mediates neural input from the vestibular nuclei to the spinal motoneuron (Wilson 1993). Hence, the IMC at 8–12 Hz is considered to reflect the neural input form the reticular formation and vestibular nuclei to the spinal motoneuron. In addition, it is suggested that aroused emotions activate the amygdala, which is related to autonomic nervous activity, and send excitatory commands to the reticular formation and vestibular nuclei (Lang et al. 2000; Balaban and Thayer 2001; Balaban 2002; Staab et al. 2013). In the present study, therefore, aroused emotions may have increased common neural input from the reticular formation and vestibular nuclei to spinal motoneurons between ankle plantarflexors. Moreover, since the spinal gamma motoneuron receives neural input via the reticulospinal tract, this further supports the above-mentioned possibility that aroused emotions increased common neural input to the muscle spindle (Hulliger 1984).

### Modulation of synchronized neuromuscular activity at 15–30 and 30–40 Hz between ankle plantarflexors in aroused emotions

In the present study, significant IMCs were observed at 15–40 Hz across all conditions and muscle pairs, although their values were relatively weak (Fig. 6B). This finding is consistent with a previous study showing cortical contributions to postural control during quiet standing (Jacobs et al. 2015). Furthermore, IMCs in the SOL-MG and SOL-LG pairs were increased at 15–30 Hz under aroused emotional conditions (Fig. 6B). Beta-band IMC is well known to reflect common neural inputs to different motoneuron pools originating from the motor cortex (Norton and Gorassini 2006; Fisher et al. 2012). Thus, our findings suggest that synchronized activity between the ankle plantarflexors, which originates from the cortical motor areas, is enhanced in aroused emotional states. This result aligns with a previous finding that beta-band IMC is increased with initial exposure to postural threat (i.e., arousal increase) and is then decreased with repeated exposure to the threat, along with reduced arousal levels (Zaback et al. 2022). This further supports the relationship between arousal states and synchronized ankle plantarflexor activity originating from the motor cortex. Additionally, emotionally arousing images have been reported to increase corticospinal excitability, regardless of valence (Hajcak et al. 2007). Given the strong correlation between beta-band IMC and corticospinal excitability (Power et al. 2006), it is possible that the corticospinal excitability of the ankle plantarflexors is enhanced in aroused emotional states during quiet standing.

In contrast to IMC value at 15–30 Hz, IMC at 30–40 Hz was significantly decreased in aroused emotional conditions (Fig. 6B). Higher IMC values in the gamma band have been reported to be associated with dynamic movement (Semmler et al. 2002; von Tscharner 2013). In the present study, IMC at 30–40 Hz was significantly positively correlated with SD-ML (i.e., amplitude of COP), although no significant effect of arousal on SD-ML was found (Table 1; Fig. 3; Table 2). This suggests that decreased IMC in gamma band under aroused emotional conditions may be partially related to reduction in the dynamic adjustment of ankle torque for postural control. Additionally, previous studies have reported that IMC values in gamma band are increased during novel and complex movements that require attentional focus (De Marchis et al. 2015; Jensen et al. 2018; von Tscharner et al. 2018). When participants are viewing arousing pictures, attentional resources are directed toward visual information (Bradley et al. 2006; Leite et al. 2012). Given that cognitive task during quiet standing shift attentional resources away from postural control, leading to more automatic postural control (Rosso et al. 2017), the decreased IMC value at 30–40 Hz may be related to allocation of attentional resources to the processing of viewing arousing pictures and the suppression of attention toward postural control. This may further be supported by the current result of positive correlation between IMC at 30–40 Hz and SD-ML because COP amplitude is decreased with the suppressive attention toward postural control (Donker et al. 2007).

### Absence of the correlation between MPF-AP and neuromuscular activities of ankle plantarflexors

The COP results indicated that only MPF-AP was significantly increased in aroused emotions (Fig. 3), which aligns completely with the findings of Horslen and Carpenter (Horslen and Carpenter 2011). In contrast, our previous work found that MPF-AP increased in response to unpleasant emotions, paralleling valence levels (Takahashi et al. 2024). The current study confirmed successful manipulation of both arousal and valence using EDA and ECG, whereas our previous work recorded only ECG and demonstrated successful manipulation of valence but did not provide objective information on arousal manipulation (Takahashi et al. 2024). Additionally, our prior study used emotional pictures (e.g., mutilation and dead bodies) known to elicit high arousal state in both High- and Low-Unpleasant conditions, which may have artificially induced high arousal in unpleasant conditions (Bradley et al. 2001). Therefore, these factors leave the possibility that arousal effects have been masked within the valence-driven COP modulation in the previous study (Takahashi et al. 2024) and likely explain the difference between our previous findings and the current study, as well as Horslen and Carpenter’s study (Horslen and Carpenter 2011). Height-induced postural threat elicits aroused emotions and leads to several changes in COP variables, such as increased MPF-AP, reduced SD-AP, and backward COP leaning (Adkin and Carpenter 2018). Notably, the habituation of arousal by repeated height exposure is associated with a reduction of MPF-AP, indicating that MPF-AP is susceptible to arousal (Zaback et al. 2019, 2021). However, there was no significant correlation between MPF-AP and either individual or synchronized neuromuscular activity of ankle plantarflexors (Table 2). Several factors could explain this lack of correlation. First, neuromuscular activity is transmitted to mechanical output via tendon and joint in a non-linear manner, resulting in low-frequency joint torque (Fukunaga et al. 2001; Kawakami et al. 2002). This suggests that the neuromuscular activity of ankle plantarflexors transmits mechanical torque, reflected as COP, in a non-linear fashion. In fact, more than 90% of the COP power in the AP direction is below 0.5 Hz, which complicates the identification of a direct relationship between MPF-AP and the EMG component at 0–12 Hz (Carpenter et al. 2001b). Second, COP (i.e., ankle torque) is adjusted by not only neuromuscular activity but also intrinsic mechanical properties, such as the stiffness and viscosity of the muscle-tendon unit around the ankle (Loram and Lakie 2002; Casadio et al. 2005; Loram et al. 2007). Specifically, Loram and Lakie reported that intrinsic stiffness provides approximately 90% of ankle torque necessary for stabilizing quiet standing (Loram and Lakie 2002).

### Functional implication for postural control ability

The present findings on the neurophysiological modulation of ankle plantarflexors and the change in COP variables in aroused emotions suggest a potential reduction in standing balance and an increased risk of falling as arousal levels rise. Physiological tremor, augmented through synchronized neuromuscular activity between synergist muscles at 8–12 Hz, increases force fluctuation in a force-matching task, worsening the accuracy of force adjustment (McAuley and Marsden 2000; Boonstra et al. 2008b; van der Stouwe et al. 2015; Flood et al. 2019; Laine and Valero-Cuevas 2020). Additionally, physiological tremor affects force adjustment accuracy more in low-force exertion than in high-force exertion (Novak and Newell 2017). Given that the EMG amplitude of ankle plantarflexors during quiet standing is below 10% of maximum voluntary force (Panzer et al. 1995; Kouzaki et al. 2007), aroused emotions may have prevented accurate adjustment of ankle joint torque. Besides, elderly individuals who show high risk of falling exhibited increased IMC at 8–12 Hz between ankle plantarflexors and physiological tremor of ankle plantarflexors compared to young individuals (Kouzaki and Masani 2012; Obata et al. 2014). There is also a significant positive correlation between age and MPF-AP of the COP that was increased in aroused emotions in the present study (Sasagawa et al. 2024). These findings may support the idea that aroused emotions can elevate the risk of falling.

### Limitations

There are several limitations in the present study. First, we attempted to minimize cross-talk between different EMG signals for IMC estimation. To reduce the cross-talk effect, we tried to put EMG electrodes for each muscle within the ankle plantarflexors more than 10 cm apart from those for the other two ankle plantarflexors (Hansen et al. 2005). It has been reported that when IMC is contaminated with cross-talk, high IMC values—well above the confidence limit—are observed across a broad frequency range (Hansen et al. 2005; Watanabe et al. 2018). In our study, we calculated IMC up to 450 Hz and confirmed that IMC above 40 Hz was generally low, intermittently reaching the confidence limit, while continuous significant IMC was observed below 40 Hz (Supplementary Figure). Nevertheless, given the anatomical positioning of MG and LG above SOL, potential cross-talk effects on IMC values should still be considered. Second, the present study recruited only male participants and did not include female participants. Interoception, the process of monitoring internal bodily states such as organ activity (Craig 2002), is associated with emotional response and body posture (Prentice et al. 2022; Dohata et al. 2025). Notably, interoception in females is influenced by the menstrual cycle (Prentice et al. 2022). To eliminate this potential confounding factor, we selectively recruited male participants. However, given gender differences in physiological responses to emotional stimuli (Gomez et al. 2016), caution is warranted when generalizing the present findings to female populations. Future studies should include female participants to examine potential gender differences. Third, the present study is that while measuring the EMG of ankle plantarflexors and the COP provides insights into the ankle strategy for postural control (Masani et al. 2003), it does not account for the role of the knee and hip joints in postural control. Recent studies have suggested that knee and hip joint movements play a significant role, even during quiet standing (Sasagawa et al. 2009a; Yamamoto et al. 2015). Additionally, each of ankle and hip remote torques reduces net angular acceleration of the other joint, indicating the existence of a kinetic interaction between the ankle and hip joints (Sasagawa et al. 2014). Therefore, future studies focusing on kinematics, kinetics, and neuromuscular activity around the knee and hip joints, as well as their interaction, would provide a more comprehensive understanding of the association between emotional states and standing postural control.

## Conclusion

In conclusion, aroused emotions reduce the individual neuromuscular activity of ankle plantarflexors (specifically SOL). On the other hand, aroused emotions increase synchronized neuromuscular activity within the frequency band associated with physiological tremor between ankle plantarflexors, suggesting a reduced ability for postural adjustment with aroused emotions. In addition, synchronized neuromuscular activity which originates from cortical cortex is modulated in aroused emotions. These findings enhance our understanding of the emotional effects on postural control.

## Appendix A

1. High-Pleasant: 4225, 4232, 4255, 4310, 4611, 4647, 4652, 4656, 4681, 4687, 4800, 4810;
2. High-Unpleasant: 3000, 3010, 3053, 3060, 3068, 3069, 3071, 3080, 3110, 3130, 3150, 3400;
3. Low-Pleasant: 1441, 1750, 2040, 2260, 2311, 2332, 2530, 2550, 2660, 5594, 5631, 5780;
4. Low-Unpleasant: 2095, 2141, 2205, 2276, 2375, 2590, 2750, 2900, 9000, 9220, 9330, 9331;

## Electronic supplementary material

Below is the link to the electronic supplementary material.

Supplementary Material 1

## Data Availability

No datasets were generated or analysed during the current study.
