# Supplementary material for: Modulation of individual and synchronized activities of ankle plantarflexors during quiet standing in aroused emotions
Source: Exp Brain Res. 2025 Apr 22;243(5):126. doi: 10.1007/s00221-025-07046-3 (PMC12014843; doi:10.1007/s00221-025-07046-3)
Supplement: Supplementary file 1 — Supplementary Material 1 [file 221_2025_7046_MOESM1_ESM.docx]

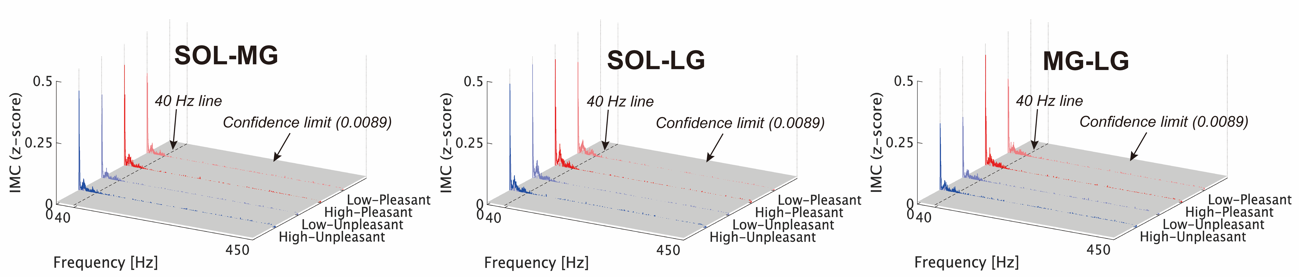


**Supplementary figure.** Pooled IMC up to 450 Hz across participants. Gray plane indicates the confidence limit value, and dotted line on it indicates 40 Hz.
